# Areca nut extract demonstrated apoptosis-inducing mechanism by increased caspase-3 activities on oral squamous cell carcinoma

**DOI:** 10.12688/f1000research.14856.5

**Published:** 2019-07-01

**Authors:** Liza Meutia Sari, Gus Permana Subita, Elza Ibrahim Auerkari

**Affiliations:** 1 Oral Medicine Department, Faculty of Dentistry, University of Syiah Kuala, Banda Aceh, 23111, Indonesia; 2 Oral Medicine Department, Faculty of Dentistry, University of Indonesia, Jakarta, 10430, Indonesia; 3 Oral Biology Department, Faculty of Dentistry, University of Indonesia, Jakarta, 10430, Indonesia

**Keywords:** Areca nut, oral cancer, apoptosis, caspase-3

## Abstract

**Background: **Oral squamous cell carcinoma is a neoplasm of keratinocyte cells of oral mucosa epithelium that can potentially spread through lymphatic tissue or blood vessel. Although areca nut is one of the plants with a risk of inducing that cancer, areca nut is believed to have high antioxidant properties. Due to the current interest in the apoptosis effects from areca nut for oral cancer treatment, we investigated its ability to induce apoptosis and caspase-3 activity in oral cancer cell lines: HSC-2 and HSC-3.

**Methods: **We examined the effect of areca nut on apoptosis and caspase-3 activity in HSC-2 and HSC-3 cells. Flow cytometry was conducted for the quantification of the cells that were apoptotic and expressing the caspase-3 enzyme for 24 and 48 hours.

**Results: **Areca nut induced a significant increase (p<0.01) in late apoptosis of HSC-2 cells and mostly occurred over 48 hours. The study also found that in HSC-3, there were significant increases (p<0.01) the percentage of cells in early apoptosis after 24 hours and late apoptosis at 48 hours. Caspase-3 activity increased after 24 and 48 hours of areca nut exposure in both cells.

**Conclusions: **The study showed that areca nut could be considered as a potential anticancer agent through its capability in inducing a caspase-dependent apoptosis.

## Abbreviations

DNA, Deoxyribose Nucleic Acid; AIF, Apoptotic Inducing factor; AP-1, Activator Protein-1; Bcl-2, B-cell lymphoma-2; COX-2, Cyclooxygenase-2; DISC, Death Inducing Signal Complex; EGF, Epidermal Growth Factor; FDA, Food and Drug Administration; FITC, Fluorescein Isothiocyanate; IC50, Inhibition Concentration 50; IGF-1, Insulin Growth Factor-1; MAPKs, Mitogen-Activated Protein Kinases; PI, Propidium Iodide; PS, Phosphatidylserine; WHO, World Health Organization.

## Background

Cancer originates from a multistep process which is modulated by environmental and genetic factors ^1^. Cancer cells undergo pathologic proliferation and no longer respond to expression signals from tumor suppressor genes, causing disruption of cell cycle phases which acts to repair DNA and eventually become antiapoptotic cells ^1, 2^. Cell cycle inhibition and apoptosis induction are two strategies in treating cancer which is considered forms of targeted therapy ^3^. Cancer cells lose the ability to control these two mechanisms ^4^. The ability of an anti-neoplastic drug is to induce cell cycle inhibition and apoptosis highly influences its potency as a cytotoxic agent. An effective chemopreventive agent should preferably interfere early in the process of carcinogenesis to eliminate premalignant cells before they acquire malignant character ^5^. Apoptosis is the process of programmed cell death and is dependent on cysteine protease enzymes called caspases ^6^. There are two pathways involved in the initiation of apoptosis, the intrinsic and extrinsic pathway ^7^. These two pathways ultimately lead to the activation of executioner caspases, caspases 3, 6, and 7 ^7^. Expression of caspase-3 is significantly lower in tumor tissue compared with normal tissue and tissue surrounding the tumor ^8^. The caspase-3 is a key effector caspase in the apoptotic program of cell suicide. The lack of caspase-3 expression may lead survival of cancer cell so that it will increase the severity of cancer.

Natural compounds are important in the treatment of life-threatening conditions. In many surveys, herbal medicines are amongst the most commonly used group of treatment. Herbal remedies are believed by the general public to be safe, cause fewer side effects and less likely to cause dependency. According to WHO, poverty and poor access to treatment cause approximately 65%–80% of world population living in developing countries to still depend on natural ingredients of plants for medicine as they are much more affordable ^9^.

Development of herbal drugs internationally has increased rapidly, with China, Europe, and the United States as the largest suppliers. The percentage of herbal drug users has reached 90% in Ethiopia, 70% in India and Chile, and 40% in China and Colombia ^10^. One study found that four in ten adults in the United States currently uses traditional alternative treatment ^11^. Of the drugs approved by the US FDA since 1984–1994, 60% are isolated from plants ^12^. Of the 121 types of drugs prescribed for cancer treatment, 90 are derived from medicinal plants ^10^.

One study reports that of the 65 new drugs listed for cancer treatment since 1981–2002, 48 originated from natural products derived from plants ^13^. Research and development of herbal medicines are needed to produce drugs which can be approved by formal health care agencies, especially in terms of their quality, safety, and efficacy ^14^.

One of the plants with potential to be developed as a herbal medicine is the *pinang* plant ( *Areca catechu* Linn; *areca*, *Palmaceae*). Indians and Malaysians chew this seed to refresh breath, smooth digestion, increase libido, treat helminth infections, and maintain stamina ^15^. Areca nut is believed to be able to induce euphoria, a tranquilized condition, with warm and comforting effects. The activities of areca nut effects include antioxidant and antihelmintic ^16– 24^, antidiabetic ^25^, antidepressant ^20^, antifungal ^24^, antibacterial ^26^, antimicrobial ^27^, antimalarial ^28^, anti-inflammatory ^22^, insecticide, psychoactive, hepatoprotective ^29^, and larvicidal ^30^, antiaging and cosmetic ^31^, hypolipidaemic ^32^ and hypoglycemic ^33^. Other studies, however, have identified negative effect from excessive areca nut consumption specifically carcinogenic properties which can induce oral squamous cell carcinoma (OSCC) ^34^. Areca nut is traditionally masticated either alone or along with a large variety of ingredients, such as betel leaf (family Piperaceae), Uncaria gambir, and slaked lime for traditional ceremonial cultural purposes in Indonesia. Previous study showed that the antioxidant activity of areca nut extract has the potential to prevent oxidative damage in normal cells ^16^. However, there are no current reports on the apoptotic mechanism of the areca nut extract on oral squamous carcinoma cell lines.

Hence in this study, the ability of areca nut to induce apoptosis and caspase-3 activity was evaluated and compared between two different time periods (24 and 48 hours) and two types of OSCC cell lines, human squamous carcinoma HSC-2 and HSC-3.

## Methods

### Sample preparation

The study materials were obtained from areca nuts of *pinang* plant from Aceh Besar, Indonesia, which was determined and documented by the Botanical Division of Biological Research Center LIPI Cibinong, complete with its roots, stems, leaves, flowers, and seeds in 2017.

### Extraction

The sample used was two kilograms of areca nut (gross weight). Areca nut was collected and cleansed from dirt (wet sortation), then washed with running water until clean and drained. Those seeds were dried in open air and covered from direct sunlight then continued with drying using an oven at 50°C. Dried *simplicia* (unprocessed natural ingredient) was crushed using a blender producing a powdered *simplicia* and sifted with 20 mesh sieves. The powder was macerated with 96% ethanol solvent. Around 500 grams powdered *simplicia* was put into a container, then 1 L of 96% ethanol was added, closed, and left for three days covered from sunlight, while repeatedly stirred. After three days the extract was strained, and the remaining extract then was dried. The dried extract was added to 500 mL of 96% ethanol and stirred, after acquiring all extract. The container was closed, left in a cool place and covered from sunlight for two days. The sediment was separated and liquid extract was obtained. Then the extract was evaporated using rotary evaporator at 30–40°C then concentrated again using water bath so a dense extract of areca nut would be obtained. The extract was stored in -20°C until further use. To prepare two different concentrations (IC _50_ areca nut extract on HSC-2 and HSC-3 cells were 629.50 µg/mL and 164.06 µg/mL respectively), 10 mg of the powder was first dissolved in 150 µl of DMSO (276855, Sigma-Aldrich) and diluted with complete culture medium to reach the desired dilution.

### Cell culture

The HSC-2 and HSC-3 cell lines were cultured in complete Dulbecco’s modified Eagle’s medium (D6429, Sigma-Aldrich) containing 10% FBS, nonessential amino acids, pyruvate, glutamine, and vitamins at 37°C with 5% CO _2_/95% air in a humified CO _2_ incubator. All media were also supplemented with 100 units/mL of penicillin and 100 mg/mL of streptomycin (15070063, Thermo Fisher Scientific). The above-mentioned cell lines were procured more than 6 months ago and have not been tested recently for authentication in our laboratory. The HSC-3 and HSC-2 cell lines used in this study were provided by the Oral Biological Laboratory, Faculty of Dentistry of the University of Indonesia. The HSC-2 and HSC-3 cell lines used in this study were given by the Section of Molecular Embryology, Graduate School of Medical and Dental Sciences, Tokyo Medical and Dental University. The HSC-3 cell line was derived from an oral squamous cell carcinoma of the tongue with a *p53* gene mutation, namely a 4bp insertion or change in the amino acid in the form of TAAG insertion in codon 305–306, exon 8 (JCRB0623) ^35^. The HSC-2 cell line was derived from an oral squamous cell carcinoma of the mouth with the *p53* splice intron 6 mutation (JCRB0622) ^35– 37^. Cell lines, placed in cryophilic liquid N _2_, were then moved into a 15 mL tube, then PBS (10010031, ThermoFisher Scientific) was added up to 10 mL. The thawing process started with centrifuging by using Laboratory benchtop centrifuge Liston C 2201 for 10 min at 300 × g at room temperature, the supernatant was disposed, the cell concentrate at the base of the tube (pellet) was added to 2–3 mL complete DMEM medium, and then it was pipetted to culture a plate containing 7–10 mL DMEM medium and was spread evenly. It was incubated at 37°C with a 5% CO _2_/95% air in a humified CO _2_ incubator. Media was changed by removing old medium from the culture plate by pipetting, rinsing with PBS two to three times, pouring new complete DMEM medium (around 7 – 10 mL) and then placing back into the incubator. If the cells achieved 80% confluence, then the confluence was ready to be harvested. The medium was disposed and rinsed with PBS Ca ^2+^ and Mg ^2+^ two to three times with the volume of 2 mL, then 1 mL Trypsin EDTA (59418C, Sigma-Aldrich) was added, then it was incubated for five to ten minutes. After the addition of complete DMEM (2 – 3 ml) and transferred into a 15 mL tube by pipetting, and centrifuging at 500 rpm for 10 minutes, the supernatant was discarded. The pellet was homogenized by pipetting, and the resuspended cells with the culture medium were ready to be used for experiment and cell counting with a hemocytometer. We had performed the cell viability assay previously to evaluate the percentage cytotoxicity and IC _50_ of areca nut extract after treating the HSC-2 cells for 72 hours is 629.50 µg/mL while in HSC-3 cells is 164.06 µg/mL ^38^. The protocol was approved by the Ethics Committee of the Faculty of Medicine, University of Indonesia no. 501/H2.F1/Etik/2014 in compliance with the International biosafety guidelines (WHO laboratory biosafety manual, 2004). 

### Treatment with areca nut extract

The HSC-2 and HSC-3 cells were plated at 1 × 10 ^5^ cells/well in 60 mm dishes with DMEM. Areca nut extract (629.50 µg/mL) was added for HSC-2 cells and 164.06 µg/mL for HSC-3 cells. For combination experiments, areca nut extracts were added at the same time and both were incubated for 24 and 48 h, before the preparation of cell extract or quantification of apoptosis and caspase-3 activity (see below).

### Analysis of apoptosis activity

A flow cytometry was used to analyze tubes containing cells with and without extract material after 24 and 48 hours exposures. Cultures of HSC-2 and HSC-3 cells with 1×10 ^5^ cells/mL concentration were centrifuged for five minutes with 500 rpm speed, washed with 1 mL cold PBS (10010031, ThermoFisher Scientific), and re-centrifuged for five minutes and vortexed. One hundred µl test solution containing 1×10 ^5^ cells in each tube is resuspended with binding buffer. 5 µL FITC Annexin V (556547, BD Pharmingen ^TM^) and 5 µL PI (556547, BD Pharmingen ^TM^) stains were added to these cells and incubated for 15 minutes in a dark place, analyzed by flow cytometry (BD FACS Calibur Flow cytometry System type E 34297502328, .San Jose, California, USA) and by manual gating using CellQuest software (Becton Dickinson, NJ). Gating was performed on blinded samples

### Analysis of caspase-3 activity

Cells were collected with and without areca nut extract for 24 and 48 hours, respectively. Prepared HSC-2 and HSC-3 cells (1×10 ^5^ cells/mL, 5 mL) were washed with cold PBS and resuspended with 400 µL BD Cytofix/Cytoperm ^TM^ Solution (51-6896KC, BD Pharmingen ^TM^). The procedure was begun by determining the amount of BD Perm/Wash ^TM^ buffer (51-6897KC, BD Pharmingen ^TM^) and 20 µL Rabbit anti-active caspase-3 polyclonal antibody (351-68655X, BD Pharmingen ^TM^) required so that each test was consist of 100 mL BD Perm/Wash ^TM^ buffer and 20 µL antibody. After incubation for 20 minutes on ice, cells were centrifuged and washed with BD Perm/Wash ^TM^ buffer. After that, BD Perm/Wash ^TM^ buffer was added and then the antibody is incubated for thirty minutes in room temperature. Each tube was rinsed again with 1 mL BD Perm/Wash ^TM^ buffer, re-centrifuged then added 300 µL BD Perm/Wash ^TM^ buffer.

### Statistical analysis

All data were presented as the mean ± standard deviation of triplicate parallel measurements. Statistical analysis used SPSS 10.0 and the data were analyzed with the unpaired t-test using a significance level of *p*<0.01.

## Results

### Apoptosis assay

**Figure 1.**
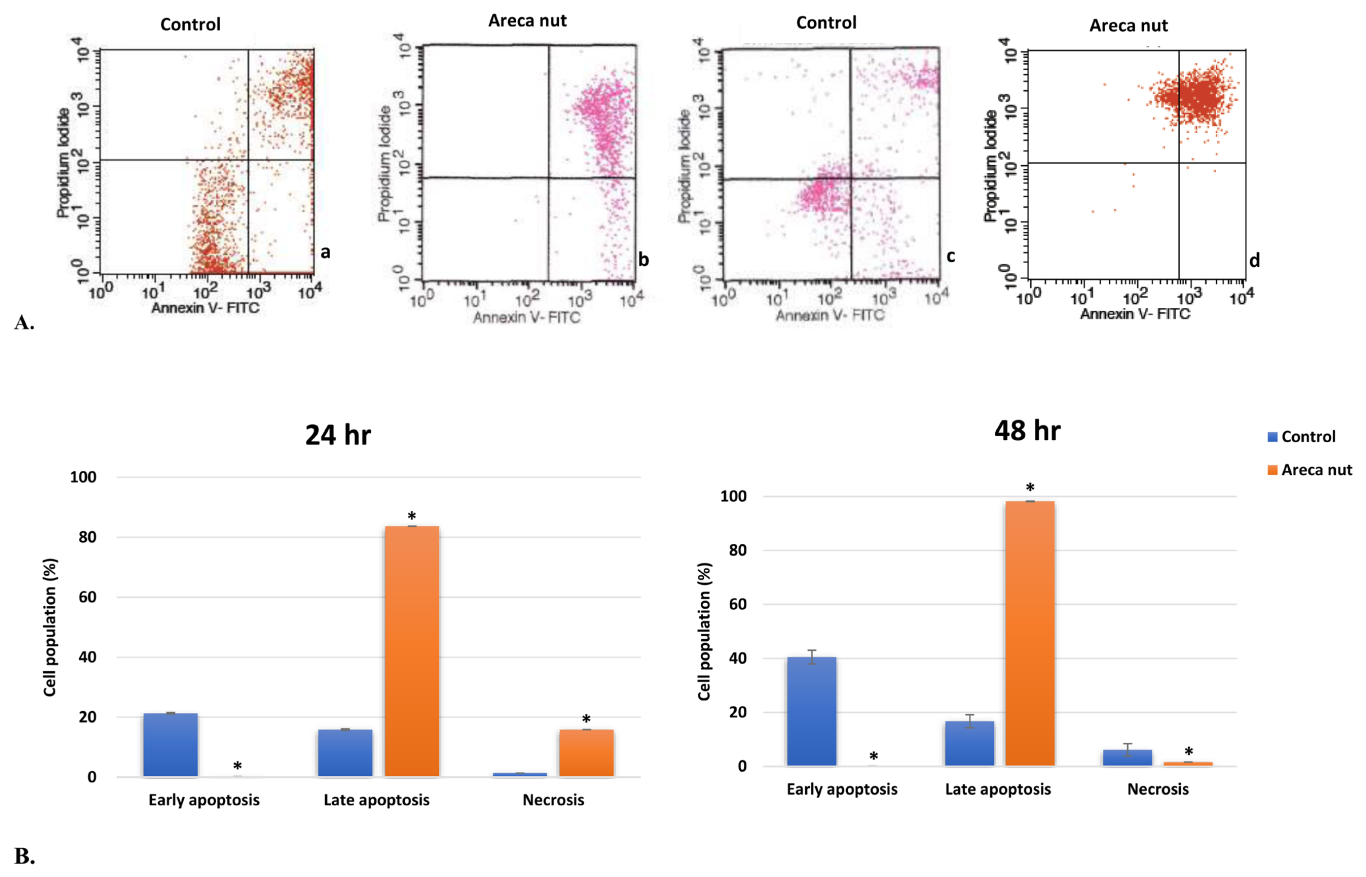
**A.** Flow cytometry analysis for apoptosis-inducing activities of areca nut on HSC-2 cells, a and b: 24 hr; c and d: 48 hr. **B**. Graph of comparison between the percentage of HSC-2 cells with and without 24 and 48 hours extract exposure at IC _50_ (629.50 µg/mL). The percentage value is mean±SD. Unpaired t-test shows the correlation of the means between control group and test group.* *p* < 0.01.

Apoptosis assay was performed on cell populations with and without areca nut extract for 24 and 48 hours. The IC _50_ dose of extract used was 629.50 μg/mL for HSC-2 cells. The percentage value of the cell population count was calculated based on the division of four quadrants, i.e. the viable cells (lower left quadrant; AV-/PI-), early apoptosis (lower right quadrant; AV+/PI-), late apoptosis (upper right quadrant; AV+/PI+), and necrotic cells (upper left quadrant; AV-/PI+). As shown in Figure 1. A.b and 1. B; left panel, apoptotic activity over 24 hours increased after areca nut extract treatment, as much as 83.73±0.02%. This number is 67.85% higher, or approximately 5.3 times higher, compared to controls (15.88±0.24%). The difference was statistically significant at p < 0.01. This increase was significant, suggesting that a reduction of viability represents mostly apoptosis.

Then, we examined the effect of areca nut extract after 48 hours of exposure. The result showed that areca nut also induced an increase in late apoptotic cells after 48 hours. As can be seen in Figure 1. A. The late apoptotic cells with red dots in the upper right quadrant indicate that areca nut has high cytotoxicity. Therefore, it can be concluded that areca nut extract is capable of inducing apoptosis in HSC-2 cells. Graphs showing a comparison of mean percentage between control cells and after areca nut extract exposure is shown in Figure 1. B; right panel.

**Figure 2.**
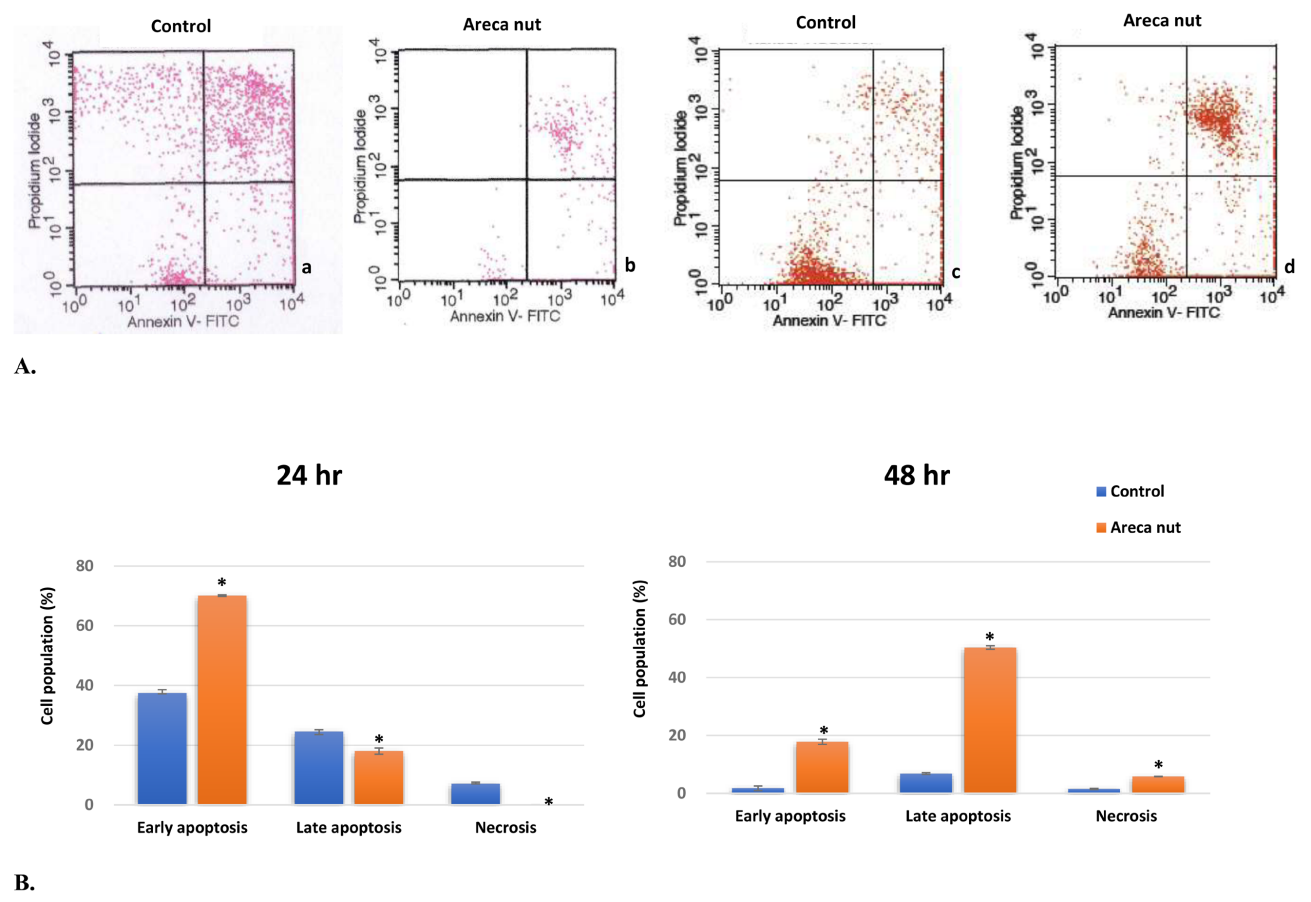
**A.** Flow cytometry analysis for apoptosis-inducing activities of areca nut on HSC-3 cells, a and b: 24 hr; c and d: 48 hr. **B.** Graph of comparison between HSC-3 cell percentage with and without 24 and 48 hours areca nut extract exposure at IC _50_ (164.06 µg/mL). The percentage value is mean±SD. Unpaired t-test shows the correlation of the means between control group and test group.* *p* < 0.01.

Although areca nut extract exposure can increase the number of late apoptosis cells more than 80% in HSC-2 cells, the viability of HSC-2 cells without areca nut extract exposure in 48 hours was 34.12% ± 2.60 %, p < 0.01 ( Figure 1. A. c). It means that the viability of the HSC-2 cells without areca nut exposure which was less than 40% resulted in a greater proportion of apoptotic cells. In our opinion, the incidence of apoptotic cells did not fully influenced by the areca nut extract exposure, but also from the small number of the viable cells. The same condition was also seen on HSC-3 cells in 24 hours, where the viability of HSC-3 cells without areca nut extract exposure was only less than 30% (27.48% ± 0.67%, p < 0.01) ( Figure 2. A. a).

The apoptosis assay performed in HSC-3 cells demonstrates a different result to that of HSC-2 cells after areca nut extract exposure for 24 hours. There was no increase in late apoptosis but, instead, the early apoptotic cell population increased (37.48% to 70.10%, p < 0.01) ( Figure 2. B; left panel). However, cells treated for 48 hours showed a significant increases in early and late apoptotic cell percentage (18.20 ± 1.03% and 70.1 ± 0.25%, p < 0.01, respectively) ( Figure 2.B; right panel). 

### Caspase-3 assay

**Figure 3.**
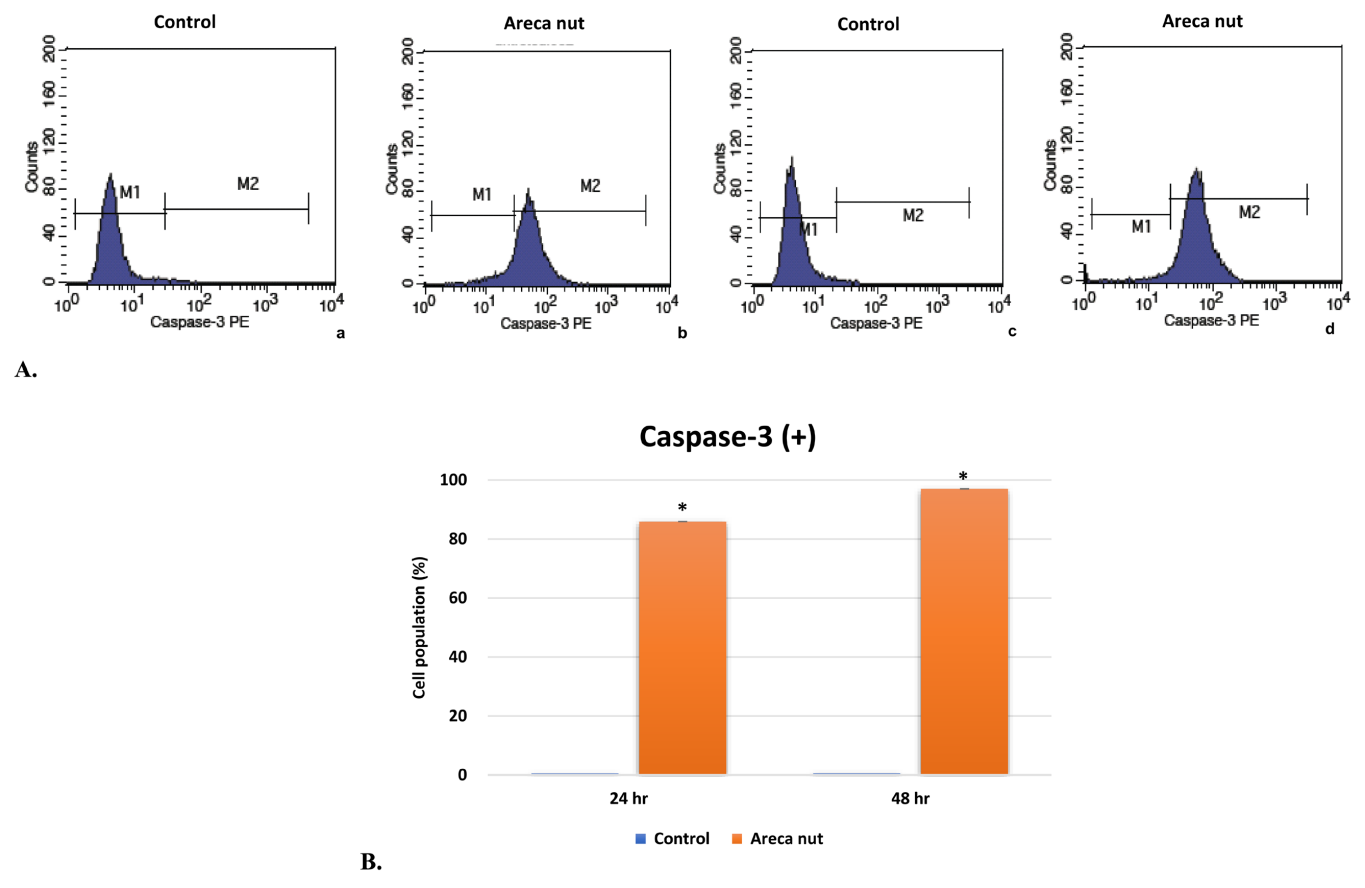
**A.** Flow cytometry analysis for caspase-3 activity inducing activities of areca nut on HSC-2 cells, a and b: 24 hr; c and d: 48 hr. **B.** Graph of comparison between the percentage of HSC-2 cells with active caspase-3 with and without areca nut extract exposure after 24 and 48 hours at IC _50_ (629.50 µg/mL). The percentage value is mean±SD. Unpaired t-test shows the correlation of the means between control group and test group.* *p* < 0.01.

We examined whether the areca nut extract can induce the activity of caspase-3 activity by using flow cytometry. The values were calculated based on the percentage of the cell population with caspase-3 enzyme activity during apoptosis. The percentage of control and test cells in the same quadrant were compared. The M _1_ quadrant demonstrates the number of living cells without active caspase-3, whereas M _2_ quadrant is a number of apoptotic cells with active caspase-3 ( Figure 3A). The results indicate that the areca nut extract can increase the number of HSC-2 cells with active caspase-3 significantly after 24 and 48 hours exposure (85.93±0.01% and 97.05±0.01%, *p* < 0.01, respectively) ( Figure 3B). The untreated cells (M _1_) were primarily negative for the presence of active caspase-3, whereas more than one-third of the treated cells were positive for active caspase-3 staining (M _2_). These values are in accordance with the results of the apoptosis test, as an increase in caspase-3 corresponds with an increase of the late apoptosis cell population.

**Figure 4.**
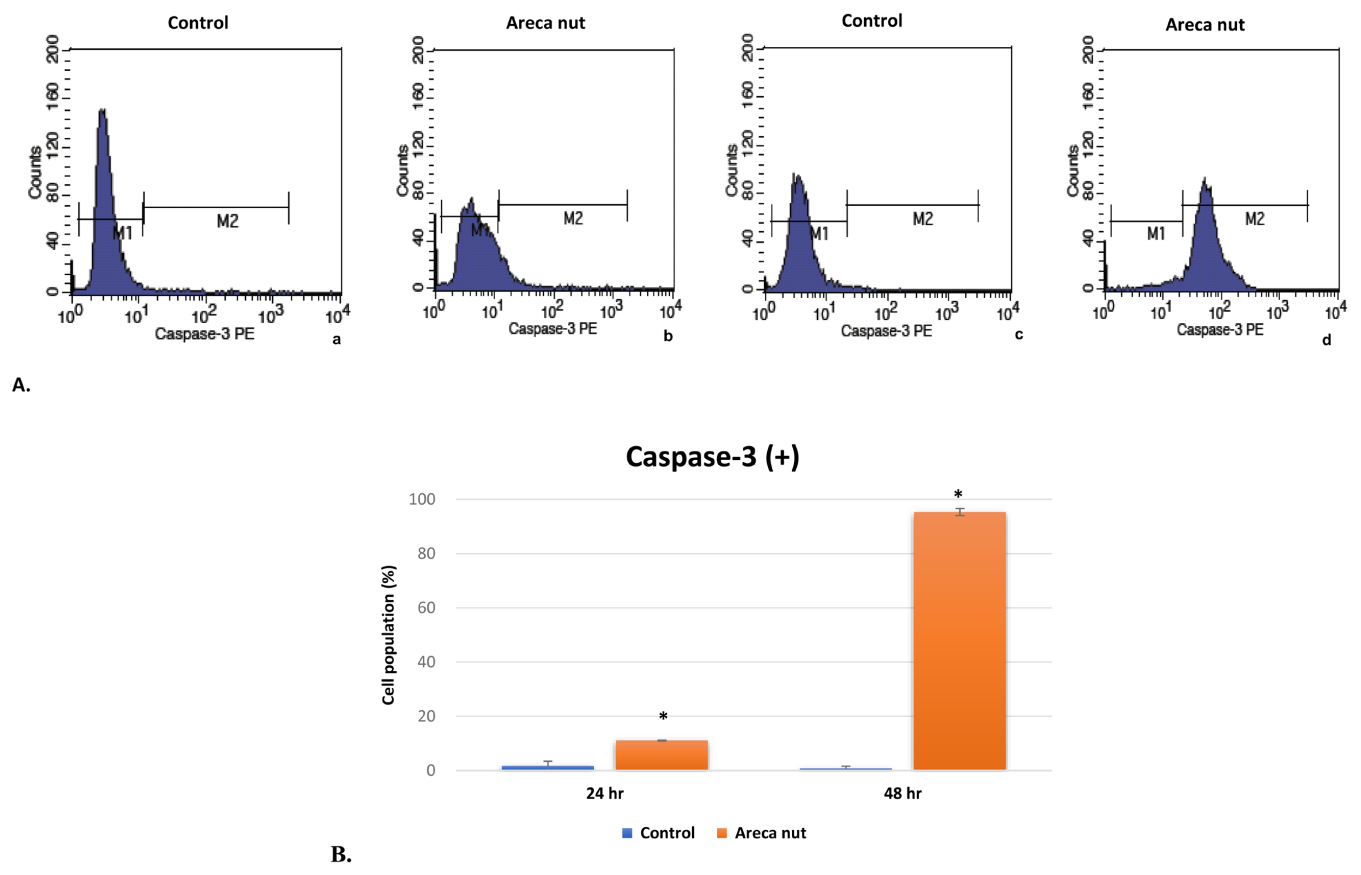
**A.** Flow cytometry analysis for caspase-3 activity inducing activities of areca nut on HSC-3 cells, a and b: 24 hr; c and d: 24 hr. **B.** Graph of comparison between the percentage of HSC-3 cells with active caspase-3 with and without areca nut extract exposure after *2*4 and 48 hours at IC _50_ (164.06 µg/mL). The percentage value is mean±SD. Unpaired t-test shows the correlation of the means between control group and test group. * *p* < 0.01.

Next, the high number of HSC-3 cells with active caspase-3, was seen increasing 8.61 times higher than control cells after 48 hours of exposure ( Figure 4B). Population distribution is also clearly shown between cells with and without extract exposure.

Output flow cytometry files for all experiments with statistical analysis output files Click here for additional data file. Copyright: © 2019 Sari LM et al. 2019 Data associated with the article are available under the terms of the Creative Commons Zero "No rights reserved" data waiver (CC0 1.0 Public domain dedication).

## Discussion

This study is a novel or first study which clearly reveals the potential cytotoxicity effect and mechanism of action of areca nut in oral squamous cell lines. Our preliminary study showed that the areca nut has a high content of total phenolic and flavonoid compounds, and showed cytotoxicity activity against HSC-2 and HSC-3 cells ^38^. In the present study, we found that areca nut extract can induce apoptosis in HSC-2 and HSC-3 cells with optimum time after 48 hours exposure. This result correlates with the previous finding that the ethanolic extract of areca nut treatment (IC _50_ 77 μg/mL) for 48 hours could inhibit the growth of MCF-7 cells ^39^.

Although the extract has the same optimum time in both cells, we found that HSC-3 cells have a greater ability to withstand apoptosis than HSC-2 cells. This result is possible because of the characteristic of HSC-3 cells is different from HSC-2 cells. Both cells have mutated *p53* tumor-suppressor gene ^35, 40^. The HSC-3 cell line has high metastatic potential with a longer doubling time than HSC-2. The HSC-2 cell line has neither invasive nor metastatic potential. However, we do not fully understand the different mechanism that occured between the two cell types in response to the areca nut. Further studies to investigate this issue will be needed.

Analysis of apoptotic cells using flow cytometry demonstrated that the viabilities of the HSC-2 and HSC-3 cells without areca nut exposure in 48 and 24 hours respectively are low (less than 40%). This condition suggests that the preparation of the staining process in flow cytometry itself may trigger the death of the cells (apoptosis or necrosis). Flow cytometry analysis was performed to identify the loss of plasma membrane asymmetry in cells. In apoptotic cells, the membrane phospholipid phosphatidylserine (PS) is translocated from the inner to the outer leaflet of the plasma membrane, thereby exposing PS to the external cellular environment. The cells are processed with enzymatic degradation, centrifugation, and/or filtration to isolate the cells of interest, and the resulting cellular suspension is “stained” with fluorescent antibodies. The small number of viable cells without areca nut extract exposure limits the conclusions that can be drawn from our results. To ascertain the biological mechanism underlying areca nut-induced death, apoptotic cell images should be obtained with a fluorescent microscope.

The present study demonstrated that the caspase-3 activity as an effector caspase is shown to be related with late apoptosis activity because of the increase of caspase-3 with increasing late apoptotic cells percentage in both cells. Analysis of caspase activity confirmed that apoptosis might be the major mechanism of cell death induced by areca nut. As far as we know, there is no similar report regarding the caspase-3 activity induced by areca nut extract, but this result is similar to several previous studies that used plants containing flavonoids to increase caspase activity in cancer cells ^41– 43^. This finding may have biological implications in cancer treatment. The caspases inside cells are in an inactive form (procaspase), but activation induces the production of other caspases leading to cell death through proteolytic activity ^44, 45^. Caspase-3 activation is a crucial component in the apoptotic signaling cascade. Based on the results obtained from our study, the apoptosis pathway involved in areca nut-induced cell death in both cancer cell lines may be through the extrinsic and intrinsic pathways. Further investigation is needed to clarify the exact mechanism through which areca nut induces apoptosis.

## Conclusion

Apoptosis is the main cell death mechanism in HSC-2 and HSC-3 cells after areca nut extract exposure for 24 and 48 hours. This is shown by the high population of early and late apoptotic cells in HSC-2 and HSC-3 cells compared to cells without extract exposure. The optimum time of apoptosis occurrence after areca nut extract is 48 hours. We postulated that one of the possible action for the apoptosis effects of this extract occurred through increased activities of the caspase-3 enzyme. This is indicated by the high activity of caspase-3 in HSC-2 and HSC-3 cells compared to cells without extract exposure, which also indicates cell death that occurred was late apoptosis. There is great potential to develop areca nut as an adjuvant chemotherapeutic agent for oral squamous cell carcinoma treatment, hence additional studies are needed, particularly *in vivo* studies to further evaluate the observed effect.

## Data availability

The data referenced by this article are under copyright with the following copyright statement: Copyright: © 2019 Sari LM et al.

Data associated with the article are available under the terms of the Creative Commons Zero "No rights reserved" data waiver (CC0 1.0 Public domain dedication). 

Dataset 1: Output flow cytometry files for all experiments with statistical analysis output files 10.5256/f1000research.14856.d206338 ^46^.
