# Supplementary material for: Areca nut extract demonstrated apoptosis-inducing mechanism by increased caspase-3 activities on oral squamous cell carcinoma
Source: F1000Res. 2019 Jul 1;7:723. Originally published 2018 Jun 11. [Version 5] doi: 10.12688/f1000research.14856.5 (PMC6668048; doi:10.12688/f1000research.14856.5)
Supplement: Peer review history

# Peer review: Reviewer response for version 5

**DOI:** 10.5256/f1000research.21549.r50623

**Published:** 2019-07-29

**Authors:** Masa-Aki Ikeda

**Affiliations:** 1 Graduate School of Medical and Dental Sciences, Tokyo Medical and Dental University, Tokyo, Japan

The revised manuscript by Sari *et al*. was improved well and adequately answered to the reviewer's comments.

I confirm that I have read this submission and believe that I have an appropriate level of expertise to confirm that it is of an acceptable scientific standard.

# Peer review: Reviewer response for version 4

**DOI:** 10.5256/f1000research.19932.r44331

**Published:** 2019-04-10

**Authors:** Masa-Aki Ikeda

**Affiliations:** 1 Graduate School of Medical and Dental Sciences, Tokyo Medical and Dental University, Tokyo, Japan

The revised manuscript by Irna et al. was improved, but there is still a lot of room for improvement as follows:

**Methods**

 Page 4

 Cell culture

 In lines 18 – 20, the authors mention that HSC-2 is the cell line without the p53 gene mutation. However, it is a misunderstanding of the authors. It has been reported that HSC-2 has p53 gene mutation.

 Sakai E, Tsuchida N.: Most human squamous cell carcinomas in the oral cavity contain mutated p53 tumor-suppressor genes ^1^.

 Page 5

 Results

 First paragraph:

 In lines 1 – 8, the authors should state that the results of this paragraph are shown in Figure 1Aa and b.

 In lines 8 – 14, the authors should also indicate that the results of this paragraph are shown in Figure 1B, left panel.

 In lines 8 – 14, the authors mentioned that the percentage late apoptotic cell number 24 hours after areca nut treatment is 83.82±15.86% and 15.54±23.52%. However, the width of the error bars in the corresponding graph (Figure 1B, left panel) does not reflect the standard deviation (i.e. ±15.86% and ±23.52%).

 Second paragraph:

 In line 4, Figure 1 should be changed to Figure 1A.

 In line 4, is there any difference between pink and red dots?

 In line 9, Figure 1 should be changed to Figure 1B.

 Third paragraph:

 In lines 3-6, the authors mentioned that, after 24 hr areca nut treatment, there was an increase in early apoptotic cell populations from untreated to treated cells (1.77% to 17.88%, respectively). However, this description does not match the graph of Figure 2B, left panel, in which the percentage of early apoptotic cells of untreated and treated cells appears to be about 40% and 70%, respectively.

 Fourth paragraph:

 In lines 1 – 3, do the authors repeat the description of the previous paragraph (Figure 2B)? If so, the authors need to eliminate redundancy. The authors state that in HSC-3 cell lines, areca nut extract induced only early apoptosis after 24 hours. However, late apoptotic cells were apparently detected in Figure 2B, left panel. The authors need to describe the results more accurately.

 In lines 3-7, if the authors mentioned their results here, the results should be described in the past tense and indicated as (data not shown). Or if the authors intended to state general facts that occur in the process of apoptosis, such explanations are not necessary here and thus should be excluded from the text.

 Fifth paragraph, Caspase-3 assay:

 In line 2, “The value is” should be changed to “The values are”.

 In line 8 - 11, the authors sate that areca nut extract caused an increase in the number of cells with active caspase-3 which is 85.94±56.86%. The standard deviation is very large. Isn't it impossible? I am wondering if the statistical calculation is correct. While the authors state that the number of cells without activating caspase-3 (14.37±11.27% after 24 hours exposure), such cells appear to be almost 0% in Figure 3B, left panel. Again, I am wondering if the width of the error bars in Figure 3B does not reflect the standard deviations of both control and areca nut-treated cell populations.

 In line 11, “This value is” must be “These values are”?

 In lines 16 – 17, the authors state that the similar patterns were seen in 24 and 48 hours after exposure (Figure 3A). However, as they already mentioned about 24 hours above, they should change the sentence to “the similar patterns were seen in 48 hours after exposure (Figure 3Ac-d, and 3B, left panel).

 Sixth paragraph:

 In line 1, The authors sate that “the high concentration of active caspase-3”. Since the authors counted the number of caspase-3 positive cells by flow cytometry, “concentration” is inappropriate.

 As in the previous fifth paragraph, the authors should state the number of cells with or without active caspase-3 after 48 hr.  

 Discussion:

 Since Discussion is too long and redundant, the authors should make them more concise by removing parts that are not directly related to their results in this study.

 Page 10:

 Fourth and fifth paragraphs

 The authors discuss the difference of the sensitivity of HSC-2 and HSC-3 cell lines based on the fact that HSC-3, but not HSC-2, has p53 gene mutation. However, as mentioned above, both cell lines carry p53 gene mutation. Thus, the authors should reorganize these paragraphs.

**Figures**

 Figure 1A – 4A:

 The authors should indicate when each data (i.e. a, b, c, and d) was obtained after areca nut treatment. Are (a and b) and (c and d) 24 hr and 48 hr, respectively?

 Figure 1B – 4B:

 The authors should check whether the standard deviation of each data is accurately reflected as the error bar of the corresponding graph.

I confirm that I have read this submission and believe that I have an appropriate level of expertise to confirm that it is of an acceptable scientific standard, however I have significant reservations, as outlined above.

## References

[1] : Most human squamous cell carcinomas in the oral cavity contain mutated p53 tumor-suppressor genes. *Oncogene*.1992;7(5) :927-33 1570156

# Response

**Published:** 2019-07-09

**Authors:** Liza Sari

**Affiliations:** Dentistry Faculty Syiah Kuala Indonesia, Indonesia

Dear Prof Masa Aki Ikeda,

Thank you very much for all of the correction you've made.

Here are the revisions that I've made for Version 5:

**1. Statement before revision**

Cell culture

......................

The HSC-2 cell line was also derived from an oral squamous cell carcinoma of the tongue but without the *p53* gene mutation (JCRB0622)

**Revision:**

Cell culture

.................

The HSC-2 cell line was derived from an oral squamous cell carcinoma of the mouth with the *p53* splice intron 6 mutation (JCRB0622)

**2. Statement before revision**

Results

Apoptosis assay

The results of the apoptosis assay in 24 hours showed an increase in percentage cell number after areca nut extract treatment undergoing late apoptosis, as much as 83.82±15.86%. This number is 68.28% higher compared to controls, or approximately 5.4 times higher than control (15.54±23.52%).

**Revision:**

Results

Apoptosis assay

As shown in Figure 1. A.b and 1. B; left panel, the apoptosis activity in 24 hours increased after areca nut extract treatment , as much as 83.73±0.02%. This number is 67.85% higher compared to controls, or approximately 5.3 times higher than control (15.88±0.24%).

**3. Statement before revision**

Results

Apoptosis assay

Paragraph 2

As can be seen in Figure 1, late apoptotic cells with pink and red dots in upper right quadrant indicated that areca nut was high cytotoxicity.

**Revision:**

Results

Apoptosis assay

Paragraph 2

As can be seen in Figure 1. A, the late apoptotic cells with red dots in upper right quadrant indicated that areca nut was high cytotoxicity.

**4. Statement before revision**

Results

Apoptosis assay

Graphs showing a comparison of mean percentage between control cells and after areca nut extract exposure is shown in Figure 1

**Revision:**

Results

Apoptosis assay

Graphs showing a comparison of mean percentage between control cells and after areca nut extract exposure is shown in Figure 1. B; right panel.

**5. Statement before revision**

Results

Figure 1.

**A.** Flow cytometry analysis for apoptosis-inducing activities of areca nut on HSC-2 cells, a and c: control; b and d: treated with areca nut.

**Revision:**

Results

Figure 1.

**A.** Flow cytometry analysis for apoptosis-inducing activities of areca nut on HSC-2 cells, a and b: 24 hr; c and d: 48 hr.

**6. Statement** **before revision**

Results

There was no increase in late apoptosis but, instead, the early apoptotic cell population increased. There was an increase in early apoptotic cell populations from untreated to treated cells (1.77% to 17.88%, respectively).

**Revision:**

Results

There was no increase in late apoptosis but, instead, the early apoptotic cell population increased (37.48% to 70.10%) (Figure 2. B; left panel). Next, the apoptosis assay in HSC-3 cells after 48 hours exposure, however, showed significant increases in early and late apoptotic cell percentage (18.20±1.03% and 70.1±0.25%, respectively) (Figure 2.B; right panel).

**7.** **Statement** **before revision**

Results

Figure 2.

A. Flow cytometry analysis for apoptosis-inducing activities of areca nut on HSC-3 cells, a and c: control; b and d: treated with areca nut.

**Revision:**

Results

Figure 2.

A. Flow cytometry analysis for apoptosis-inducing activities of areca nut on HSC-3 cells, a and b: 24 hr; c and d: 48 hr.

**8.** **Statement** **before revision**

Results

Caspase-3 assay

The caspase-3 assay was performed in triplicate in HSC-2 cells also using flow cytometry. The value is calculated based on the percentage of the cell population with caspase-3 enzyme activity during apoptosis. The percentage of control and test cells in the same quadrant was compared. The M1 quadrant demonstrates the number of living cells without active caspase-3, whereas M _2_ quadrant is a number of apoptotic cells with active caspase-3. Areca nut extract caused an increase in the number of cells with active caspase-3 which is 85.94±56.86% more than the number of cells without activating caspase-3 (14.37±11.27% after 24 hours exposure). This value is in accordance with the results of the apoptosis test, as an increase in caspase-3 corresponds with an increase of late apoptosis cell population. Untreated cells (M _1_) were primarily negative for the presence of active caspase-3, whereas greater than one-third of the treated cells were positive for active caspase-3 staining (M _2_). The similar patterns were seen in 24 and 48 hours after exposure ( Figure 3A). This shows that the ability of the extract to induce apoptosis is increased with longer exposure in HSC-2 cells.

**Revision:**

Results

Caspase-3 assay

We examined whether the areca nut extract can induce the activity of caspase-3 activity by using flow cytometry. The values are calculated based on the percentage of the cell population with caspase-3 enzyme activity during apoptosis. The percentage of control and test cells in the same quadrant were compared. The M _1_ quadrant demonstrates the number of living cells without active caspase-3, whereas M _2_ quadrant is a number of apoptotic cells with active caspase-3 (Figure 3A). The results indicated that the areca nut extract can increase the number of HSC-2 cells with active caspase-3 significantly after 24 and 48 hours exposure (85.93±0.01% and 97.05±0.01%, respectively) (Figure 3B). The untreated cells (M _1_) were primarily negative for the presence of active caspase-3, whereas greater than one-third of the treated cells were positive for the active caspase-3 staining (M _2_). These values are in accordance with the results of the apoptosis test, as an increase in caspase-3 corresponds with an increase of the late apoptosis cell population.

**9. Statement before revision**

Results

Figure 3.

A. Flow cytometry analysis for caspase-3 activity inducing activities of areca nut on HSC-2 cells, a and c: control; b and d: treated with areca nut.

**Revision:**

Results

Figure 3.

A. Flow cytometry analysis for caspase-3 activity inducing activities of areca nut on HSC-2 cells, a and b: 24 hr; c and d: 48 hr.

**10. Statement before revision**

Results

The high concentration of active caspase-3 activated in HSC-3 cells, which is increasing 126 times higher than control cells after 48 hours of exposure ( Figure 4). Population distribution is also clearly shown between cells with and without extract exposure.

**Revision:**

Results

Next, the high number of HSC-3 cells with active caspase-3, was seen increasing 8.61 times higher than control cells after 48 hours of exposure ( Figure 4B).

**11. Statement before revision**

Results

Figure 4.

A. Flow cytometry analysis for caspase-3 activity inducing activities of areca nut on HSC-3 cells, a and c: control; b and d: treated with areca nut.Figure 4.

**Revision:**

Results

Figure 4

A. Flow cytometry analysis for caspase-3 activity inducing activities of areca nut on HSC-3 cells, a and b: 24 hr; c and d: 24 hr.

**12. Statement before revision**

Discussion

Paragraph 1

This study is a novel or first study which clearly reveals the potential cytotoxicity effect and mechanism of action of areca nut in oral squamous cell lines. Our preliminary study showed that the areca nut has a high content of total phenolic and flavonoid ^38^ . The areca nut has chemosensitivity of cancer cells in different concentrations. We performed an MTS assay to observe the areca nut extract on cell viability. Five doses were adding into cancer cells, which were 160, 320, 640, 1280, and 2560 μg/mL in HSC-2, HSC-3, and HaCat cells ^38 ^. We found that the areca nut extract was cytotoxic towards HSC-2 (IC50 629.50 µg/mL), while in the HSC-3 cells, the IC50 is lower than HSC-2 cells (IC50 164.06 µg/mL). The areca nut showed weak cytotoxicity against HSC-2 cells. Sakagami *et al.* found that flavonoid-related phenols especially flavones showed weak cytotoxic activity against HSC-2 ^39 ^.

**Revision:**

Discussion

Paragraph 1

This study is a novel or first study which clearly reveals the potential cytotoxicity effect and mechanism of action of areca nut in oral squamous cell lines. Our preliminary study showed that the areca nut has a high content of total phenolic and flavonoid and showed cytotoxicity activity against HSC-2 and HSC-3 cells ^38^ . In the present study, we found that areca nut extract can induce apoptosis in HSC-2 and HSC-3 cells with optimum time after 48 hours exposure. This result is similar with the previous study that the ethanolic extract of areca nut (IC _50_ 77 μg/mL) for 48 hours could inhibit the growth of MCF-7 cells ^40^.

**13. Statement before revision**

Discussion

Paragraph 2

This study performs apoptosis and caspase-3 activity tests using flow cytometry, with the objective to acknowledge whether the cell death mechanism happens through apoptosis induction by areca nut extract or not. In order to acknowledge the optimum time of areca nut extract activity against the cells, two units of time are used, which are 24 and 48 hours. The results of flow cytometry analysis on HSC-2 cells shows that areca nut extract can induce late apoptosis activity after 24 and 48 hours exposure, but the increase of late apoptotic cells occurs more following 48 hours exposure. This result is in accordance with the past study that performed apoptosis test using orange acridine-ethidium bromide staining (double staining). The result showed that treatment with an ethanolic extract of areca nut (IC _50_ 77 μg/mL) for 48 hours inhibits the growth of MCF-7 cells as much as 13–84% ^40 ^.

**Revision:**

Discussion

Paragraph 2

Although the extract has the same optimum time in both cells, we found that HSC-3 cells have the ability to withstand apoptosis higher than HSC-2 cells. This result is possible because of the characteristic of HSC-3 cells is different from HSC-2 cells. Both cells have mutated *p53* tumor-suppressor gene [2, 3].The HSC-3 cell line has high metastatic potential with doubling time longer than HSC-2. The HSC-2 cell line has neither invasive nor metastatic potential. However, we do not fully understand the different mechanism that occured between the two cell types in response to the areca nut. Further studies to investigate this issue will be needed.

**14. Statement before revision**

Discussion

Paragraph 3

The flow cytometry analysis was performed to reveal the loss of plasma membrane asymmetry in cells. In apoptotic cells, the membrane phospholipid phosphatidylserine (PS) is translocated from the inner to the outer leaflet of the plasma membrane, thereby exposing PS to the external cellular environment. Annexin V is a 35–36 kDa Ca2+-dependent phospholipid-binding protein with high affinity for PS and binds to exposed apoptotic cell surface PS. Annexin V can be conjugated to fluorochromes while retaining its high affinity for PS and thus serves as a sensitive probe for flow cytometric analysis of cells undergoing apoptosis. This is one of the earliest features of apoptosis. In our research, The flow cytometry was performed triple for both cells. The cells are processed with enzymatic degradation, centrifugation, and/or filtration to isolate the cells of interest, and the resulting cellular suspension is “stained” with fluorescent antibodies. When HSC-2 cells were cultured with areca nut for 48 hours, most of the cells were in the upper right quadrant; AV+/PI+. It means that most of the cells have undergone late apoptosis ( Figure 1B). However, when HSC-2 cells were cultured for 48 hours under the same condition without areca nut treatment, we found that only less than 40% of the cells were viable. This condition suggests that the preparation of the staining process in flow cytometry itself may trigger the death of the cells (apoptosis or necrosis). The same result is seen in the HSC-3 cells for 24 hours without treatment. This includes one of the limitations of our research. To ascertain the biological mechanism underlying areca nut-induced death, the apoptotic cell images should be obtained by a fluorescent microscope.

**Revision:**

Discussion

Paragraph 3

The flow cytometry analysis was performed to reveal the loss of plasma membrane asymmetry in cells. In apoptotic cells, the membrane phospholipid phosphatidylserine (PS) is translocated from the inner to the outer leaflet of the plasma membrane, thereby exposing PS to the external cellular environment. The cells are processed with enzymatic degradation, centrifugation, and/or filtration to isolate the cells of interest, and the resulting cellular suspension is “stained” with fluorescent antibodies. In our finding, most of the treated HSC-2 cells were undergone late apoptosis ( Figure 1A). However, we found that only less than 40% of the control cells were viable. This condition suggests that the preparation of the staining process in flow cytometry itself may trigger the death of the cells (apoptosis or necrosis). The same result is seen in the untreated HSC-3 cells for 24 hours without treatment. This includes one of the limitations of our research. To ascertain the biological mechanism underlying areca nut-induced death, the apoptotic cell images should be obtained by a fluorescent microscope.

**15. Statement before revision**

Discussion

Paragraph 4

Areca nut extract can possibly induce non-apoptotic cell death or necrosis. This is shown from the increase in necrotic cell percentage significantly after 24 hours of exposure. One of the past studies using catechin from green tea, proved that catechin has the ability to induce necrosis or non-apoptotic cell death in leukemia cells without caspase-8, 9, and 3 activities ^41^ Although molecular mechanism pathway of necrosis is not clearly understood, catechin can possibly induce necrosis through two pathways, which are decreasing concentration of intracellular ATP and interaction on ATP-binding site of glucose-regulated protein (GRP78) leading to increased activity of ATPase ^41^ . This result shows two competitive abilities between catechin and ATP-binding site leading to necrosis with catechin activity via the apoptosome (intrinsic pathway) and death induced signaling pathway (DISC; extrinsic pathway).

**Revision:**

Discussion

Paragraph 4

The present study demonstrated that the caspase-3 activity as an effector caspase is shown to be related with late apoptosis activity because of the increase of caspase-3 with increasing late apoptotic cells percentage in both cells. Analysis of caspase activity confirmed that apoptosis might be the major mechanism of cell death induced by areca nut. As far as we know, there is no similar report regarding the caspase-3 activity by areca nut, but this result is similar to several previous studies that used plants containing flavonoid to increase caspase activity in cancer cells[4, 5] ^42^ . This finding may have biological implications in cancer treatment. Caspases inside cells are in an inactive form (procaspase), but activation induces the production of other caspases leading to cell death through proteolytic activity ^46^ ^, 47 ^. Caspase-3 activation is a crucial component in the apoptotic signaling cascade. Based on the results obtained from our study, the apoptosis pathway involved in areca nut-induced cell death in both cancer cell lines may be through the extrinsic and intrinsic pathways. Further investigation is needed to clarify the exact mechanism through which areca nut induces apoptosis.

**16. Statement before revision**

Discussion

Paragraph 5

Analysis of caspase-3 activity in HSC-2 cells shows results in accordance with the apoptosis assay, in that caspase-3 activity increases significantly after areca nut extract for 24 and 48 hours compared to control, with the increase of caspase-3 activity also being higher after 48 hours exposure. This result is similar to the study using catechin of green tea and hydrate catechin against HS-sultan and RPMI8226 cell strains, and MCF-7 cells using Western blot and quantitative RT-PCR techniques, that this compound can induce caspase-3, 8, and 9 activities ^42^ .

**Revision:**

Discussion

Paragraph 5

This research showed that the apoptosis activity using flow cytometry has several advantages, including fast period time analysis (thousand of cells per second), single cell analysis, and multiparametric measurements (correlations with several different cell events in one unit of time), but this machine also has drawbacks; the presence of physical and enzymatic manipulations during cell preparation and staining, can trigger additional apoptosis or necrosis cell numbers. Furthermore, flow cytometry is an efficient machine to calculate the number of apoptotic cells based on PS staining out of the cell membrane, so it is more appropriate to detect early apoptosis. If the test aims to improve the accuracy of DNA fragmentation calculations in late apoptosis, it is recommended to use *Terminal deoxynucleotidyl transferase (TdT) dUTP Nick-End Labeling (TUNEL)*.

**17. Statement before revision**

Discussion

Paragraph 6

The results of flow cytometry analysis on HSC-3 cells show that areca nut extract could also induce apoptosis after extract exposure for 24 and 48 hours. Unlike with HSC-2 cells, extract exposure induced more early apoptosis after 24 hours exposure, but after 48 exposure apoptosis induction by the extract happened more in the end step. Caspase-3 activity as an effector caspase is shown to be related with late apoptosis activity because of the increase of caspase-3 with increasing late apoptotic cells percentage. There is a significant increase in necrotic cells percentage after 48 hours of exposure. Therefore, the apoptosis assay showed that areca nut extract is capable of inducing apoptosis in HSC-2 and HSC-3 cells with an optimum time after 48 hours exposure. Although the extract has the same optimum time in both cells, there is a difference on extract effect on the number of apoptotic cells, where the percentage of HSC-2 cells undergoing apoptosis is higher than HSC-3 cells. This result is possible because of the characteristic of HSC-3 cells is different from HSC-2 cells. The HSC-3 cells have the p53 gene mutation ^36^ . The mutation of HSC-3 cells was confirmed in a previous report ^43^ . However, when the p53 gene mutates, the mutated p53 protein is excessively produced or accumulated, thereby compromising apoptosis and leading to abnormal or malignant cell growth ^44^ . We found that HSC-3 cells have the ability to withstand apoptosis higher than HSC-2 cells. However, this finding may vary by the study design and so much more data must be collected to better understand this phenomena.

**Revision:**

Discussion

Paragraph 6

***Deleted***

**18. Statement before revision**

Discussion

Paragraph 7

Literature shows that in addition to the *p53* gene mutation in HSC-3, severe damage to phosphorylation of Ser ^46^ in HSC-3 cells causes loss of apoptotic ability mediated by *p53* and also increased survival ability of HSC-3 cells against anticancer genes compared to HSC-2 cells ^45^ . The *p53* tumor suppressor gene holds an important role in deciding the cells’ fate if there is DNA damage. If there is mild damage, *p53* will stop the growth until the DNA repair process is done. If there is severe damage, *p53* will induce senescence process to prevent the increase of precancerous cells. Phosphorylation of Ser ^46^ causes *p53* to activate proapoptotic genes, leading to the induction of apoptosis ^45^ . However, the different response between HSC-2 and HSC-3 cells after exposure to extract could possibly be caused by different effects of areca nut extract on the extrinsic and intrinsic pathways. Due to the effects of the *p53* mutation on the intrinsic pathway in HSC-3 cells raises the possibility of the effects of the extracts being solely through the extrinsic pathway, whereas in HSC-2 cells, the extract works on the extrinsic and intrinsic pathways leading to more apoptosis occurring in HSC-2 cells. This cannot be determined from these results as not tests on caspase 8 and 9 were performed.

**Revision:**

Discussion

Paragraph 7

***Deleted***

**19. Statement before revision**

Discussion

Paragraph 8

The induction of apoptosis in tumor cells is considered a valuable method to treat cancer. A wide variety of natural substances have been recognized to have the ability to induce apoptosis in various tumor cells. Apoptosis is an active form of cell suicide controlled by a network of genes, in which the Bcl-2 family proteins play an important role in the control of apoptosis. The balance of pro- and anti-apoptotic Bcl-2 family proteins control permeabilization of the outer mitochondrial membrane and release of intermembrane space proteins, most notably cytochrome c. In the presence of cytochrome c and dATP, Apaf-1, the scaffold around which the apoptosome is built, recruits and activates caspase-9, which then propagates a cascade of further caspase activation events downstream.

**Revision:**

Discussion

Paragraph 8

***Deleted***

**20. Statement before revision**

Discussion

Paragraph 9

Caspases inside cells are in an inactive form (procaspase), but activation induces the production of other caspases leading to cell death through proteolytic activity ^46^ ^, 47 ^. Initiator caspase activation (caspase-8 and 9) by catechin shows early apoptotic activity in cell death. Cell death through extrinsic pathway can be influenced by catechin derivates originating from green tea, resulting in inhibition NF-kB, MAPKs signals, nitric oxide synthesis, and EGFR mediated by transduction pathway signaling through suppressing on EGF binding with its receptor, AP-1, IGF-1 signaling pathway, COX-2, and proteasome activity ^46^ . Cell death through the mitochondrial pathway can also be induced by catechin. Changes in mitochondria caused by an increase in membrane permeability lead to opened pores and loss of the mitochondrial transmembrane potential causing the release of cytochrome c into the cytosol, thereby activating the caspase-9 and 3 pathway ^46^ .

**Revision:**

Discussion

Paragraph 9

***Deleted***

**21. Statement before revision**

Discussion

Paragraph 10

Catechin can increase apoptogenic protein release from mitochondria such as cytochrome c, Smac/DIABLO, and AIF into cytosol leading to death signaling from inside of the mitochondria releasing more and activating caspase-3 ^48^ . The decrease of Bcl2 and Bcl-XL antiapoptotic protein, an increase of Bax proapoptotic protein in the intrinsic pathway are also influenced by catechin. If there is a *p53* mutation, the function of BH-3 proapoptotic protein will be inhibited and function of Bcl-2 antiapoptotic protein family will increase leading to inhibition of anticancer agent activity in the intrinsic pathway. Caspase-3 activation is a crucial component in the apoptotic signaling cascade. Based on the results obtained from our study, the apoptosis pathway involved in areca nut-induced cell death in both cancer cell lines may be through the extrinsic and intrinsic pathways. Further investigation is needed to clarify the exact mechanism through which areca nut induces apoptosis.

**Revision:**

Discussion

Paragraph 10

***Deleted***

**22. Statement before revision**

Discussion

Pararagraph 11

This research also showed that the apoptosis activity using flow cytometry has several advantages, including fast period time analysis (thousand of cells per second), single cell analysis, and multiparametric measurements (correlations with several different cell events in one unit of time), but this machine also has drawbacks; the presence of physical and enzymatic manipulations during cell preparation and staining, can trigger additional apoptosis or necrosis cell numbers. Furthermore, flow cytometry is an efficient machine to calculate the number of apoptotic cells based on PS staining out of the cell membrane, so it is more appropriate to detect early apoptosis. If the test aims to improve the accuracy of DNA fragmentation calculations in late apoptosis, it is recommended to use *Terminal deoxynucleotidyl transferase (TdT) dUTP Nick-End Labeling (TUNEL)*.

**Revision:**

Discussion

Pararagraph 11

**Paragraph 11 was removed to paragraph 5**

**23. References**

**Addition of new references**

Lin CC, Yang JS, Cheng JT, et al: **Berberine induces apoptosis in human HSC-3 oral cancer cells via simultaneous activation of the death receptor-mediated and mitochondrial pathway**. *Anticancer Res *2007, **27**:3371-3378.

Das A, Banik NL, Ray SK: **Flavonoids activated caspases for apoptosis in human glioblastoma T98G and U87MG cells but not in human normal astrocytes**. *Cancer *2010, **116**(1):164-176.

# Peer review: Reviewer response for version 3

**DOI:** 10.5256/f1000research.18953.r41638

**Published:** 2019-04-01

**Authors:** Irna Sufiawati

**Affiliations:** 1 Department of Oral Medicine, Faculty of Dentistry, Padjadjaran University, Bandung, Indonesia

I have read the new version of the article. I believe that I have an appropriate level of expertise to confirm that it is of an acceptable scientific standard.

I confirm that I have read this submission and believe that I have an appropriate level of expertise to confirm that it is of an acceptable scientific standard.

# Response

**Published:** 2019-04-04

**Authors:** Liza Sari

**Affiliations:** Dentistry Faculty Syiah Kuala Indonesia, Indonesia

Dear Dr. Irna,

 Thank you so much for your support and valuable responses.

 I'm very honored that my article was reviewed by you.

 I thank you for it and I pray that God will bless you.

 Sincerely

 Dr. Liza

# Peer review: Reviewer response for version 3

**DOI:** 10.5256/f1000research.18953.r41639

**Published:** 2019-01-21

**Authors:** Masa-Aki Ikeda

**Affiliations:** 1 Graduate School of Medical and Dental Sciences, Tokyo Medical and Dental University, Tokyo, Japan

Several improvements have been made in the revised manuscript, but there are still points to be improved.

 First, as suggested previously, very low survival rate of untreated cells in Figure 1 and 2 makes the results less convincing. For example, while approximately 80% untreated cells survived after 48 hours, only 25% of untreated cells (control) survived and more than 50% of untreated cells underwent apoptosis (early + late) after 24 hours in Figure 2B. These results are obviously weird and maybe due to inappropriate assay conditions or something else. The detected apoptosis in untreated cells is too large to be considered as “false positives”. In the case of experimental biology, variations among samples can result from biological variations or technical errors related to the method used. In general, the biological variations are larger than the technical variations. Thus, it is necessary to repeat the whole experiment independently several times. The authors stated that triplicate measurements were done in parallel in this study, but it appears that they might consider only technical variations. Carrying out independent repetition to check the reproducibility of the results is the best way to verify their findings and avoid the existence of “false positives” in their data.

 Second, if the hypothesis was supported after verifying the findings, the authors might do additional experiments to confirm it. Regarding this point, the authors have carried out experiments by using another reagent, an active caspase-3 antibody, to confirm the Annexin V results. However, their findings obtained in this study merely relies solely on one methodology, flow cytometry. Generally, experimental biology studies confirm findings by using combinations of several different methods and assays. Therefore, it would be better for the authors to verify their findings by using another method different from flow cytometry, for example, showing apoptotic cell images obtained by a fluorescent microscope.

 Minor points:

 Figures 1 and 2

 They look confusing, because each element of the graphs is not arranged as usual. It is better to exclude the bars indicating viable cell populations from the Figures. Showing only dead cell populations (early apoptosis, late apoptosis, and necrosis) appears to be sufficient, because viable cell populations indicate merely the rest of dead cell populations. Furthermore, usually the authors show the results from left to right in order of things occurring. That is, early apoptosis first on left, then late apoptosis, and necrosis on right (not very important). Finally, it would be better to reverse the order of the bars indicating Areca nut and control, i.e. control on left and Areca nut on right.

 Figure 3

 The authors should make the similar changes as mentioned as to Figures 1 and 2. First, showing only right panels, i.e. caspase-3 (+) of 24 h and 48 h, is sufficient, because left panels, i.e. caspase-3 (-), indicates only the rest of cell populations shown in right panels, i.e. these indicate the same meaning. Second, it would be better to reverse the order of the bars indicating Areca nut and control.

I confirm that I have read this submission and believe that I have an appropriate level of expertise to confirm that it is of an acceptable scientific standard, however I have significant reservations, as outlined above.

# Response

**Published:** 2019-02-07

**Authors:** Liza Sari

**Affiliations:** Dentistry Faculty Syiah Kuala Indonesia, Indonesia

1. Thank you very much for the detailed correction. At the time we do the apoptosis assay, it was true that the amount of the untreated HSC-2 cells that survived 24 hours initially accounted for ± 60% and 35% after 48 hours. The same condition was also seen in HSC-3 cells. Our opinion about this condition is that the little amount of viable cells is caused by the staining procedure. We agree with your suggestion that we need to do the different technique to confirm one test to another. We will try to fix our limitations in the next research (we’re still waiting for the grant from our university). Actually, we've also tested the cell cycle profile and Ki-67 activity by flow cytometry. The results also showed a significant increase of SubG1 phase on HSC-2 and HSC-3 after areca nut treatment.
2. Revision of the graphs: We have made the revisions of all the graphs as you instructed. We hope you’re pleased with the revisions.

# Peer review: Reviewer response for version 1

**DOI:** 10.5256/f1000research.16170.r38222

**Published:** 2018-09-25

**Authors:** Masa-Aki Ikeda

**Affiliations:** 1 Graduate School of Medical and Dental Sciences, Tokyo Medical and Dental University, Tokyo, Japan

In the present article Liza Meutia Sari *et al*. examined the effect of areca nut extract on apoptosis in oral squamous cell carcinoma cell lines, HSC-2 and HSC-3 cells. They show that areca nut extract induced apoptosis in these cells by PI and Annexin-V staining followed by flow cytometry. They confirmed the results by detecting active caspase-3 activity following areca nut extract treatment. They conclude that areca nut extract induces apoptosis and caspase-3 activity in HSC-2 and HSC-3 cell. This is a limited but well-conducted study. However, there are several points that need to be addressed.

 Specific points:

- Chemosensitivity of cancer cells is determined by adding different concentrations of a drug. The authors should present the data regarding the effect of different concentrations of areca nut extract on cell viability, when they determined the IC _50_of HSC-2 and HSC-3 cells.
- According to Abstract and Methods, the authors carried out statistical analysis of their data. They should indicate which data are statistically significantly different in each graph.
- Figure 1B, 2B, 3B, and 4B:
  The chart legends should be "Areca nut" but not its concentrations. The concentrations should be written in Figure legends.
- Figure 1B, Top:
  24 and 48 should be 24 h and 48 h, respectively.
- Figure 1B (right panel):
  It appears that many apoptotic cells (>50%) are detected and only less that 40% of cells are viable in control at 48 h, raising a question about the reliability of the results. The authors should clarify this point.
- Figure 2A and 2B (left panel):
  While more than 80% of cells are viable in control at 48 h, many apoptotic cells (>60%) are detected and only 25% of cells are viable in control at 24 h, raising a question about the reliability of the results. The authors should clarify this point.
- Figure 3B and 4B:
  By changing M1 and M2 to caspase-3 (-) and caspase-3 (+), respectively, readers will be able to understand the results more easily. 
- Discussion page 10, the middle of 1st para:
  The authors mentioned "the percentage of HSC-2 cells undergoing apoptosis is high than HSC-3 cells. This result is possibly because there is a difference of cell sensitivity againt areca nut extract". However, HSC-2 cells were treated with a higher concentration (629.5 ug/ml) of areca nut extract than HSC-3 cells (164.05 ug/ml). Because the IC50 of HSC-2 cells is higher than that of HSC-3 cells, HSC-3 cells appear to be more sensitive to areca nut extract than HSC-2 cells. This is confusing. The authors should clarify this point.

I confirm that I have read this submission and believe that I have an appropriate level of expertise to confirm that it is of an acceptable scientific standard, however I have significant reservations, as outlined above.

# Response

**Published:** 2018-10-18

**Authors:** Liza Sari

**Affiliations:** Dentistry Faculty Syiah Kuala Indonesia, Indonesia

- Chemosensitivity of cancer cells is determined by adding different concentrations of a drug. The authors should present the data regarding the effect of different concentrations of areca nut extract on cell viability, when they determined the IC _50_of HSC-2 and HSC-3 cells.
- *Comment: Our preliminary study showed that the areca nut has a high content of total phenolic and flavonoid. ^1^ The areca nut has chemosensitivity of cancer cells in different concentrations. We performed* *a MTS* *assay to observe the [G1] areca nut extract on cell viability. Five doses were adding into cancer cells, which were 160, 320, 640, 1280, and 2560 μg/mL in HSC-2, HSC-3, and HaCat cells.1 We found that the areca nut extract was cytotoxic towards HSC-2 (IC _50_ 629.50 µg/mL), while in the HSC-3 cells, the IC _50_ is lower than HSC-2 cells (IC _50_ 164.06 µg/mL). The areca nut showed weak cytotoxicity against HSC-2 cells. Sakagami et al. found that flavonoid-related phenols especially flavones showed weak cytotoxic activity against HSC-2.*

 

- According to Abstract and Methods, the authors carried out statistical analysis of their data. They should indicate which data are statistically significantly different in each graph.
- Figure 1B, 2B, 3B, and 4B:The chart legends should be "Areca nut" but not its concentrations. The concentrations should be written in Figure legends.
- Figure 1B, Top:24 and 48 should be 24 h and 48 h, respectively.
- *Comments: Thank you very much for the corrections, we have corrected all the figures as you instructed.*

 

- Figure 1B (right panel):It appears that many apoptotic cells (>50%) are detected and only less that 40% of cells are viable in control at 48 h, raising a question about the reliability of the results. The authors should clarify this point.
- *Comments:*
- *The flow cytometry analysis was performed to reveal the loss of plasma membrane asymmetry in cells. In apoptotic cells, the membrane phospholipid phosphatidylserine (PS) is translocated from the inner to the outer leaflet of the plasma membrane, thereby exposing PS to the external cellular environment.* *Annexin V is a 35–36 kDa Ca ^2+^-dependent phospholipid-binding protein with high affinity for PS and binds to exposed apoptotic cell surface PS. Annexin V can be conjugated to fluorochromes while retaining its high affinity for PS and thus serves as a sensitive probe for flow cytometric analysis of cells undergoing apoptosis[G1* *]* *.* *This is one of the earliest features of apoptosis. **In our research, The flow cytometry was performed triple for both cells. The cells are processed with enzymatic degradation, centrifugation, and/or filtration to isolate the cells of interest, and the resulting cellular suspension is “stained” with fluorescent antibodies. When HSC-2 cells were cultured with areca nut for 48 hours, most of the cells were in the upper right quadrant; AV ^+^/PI ^+^. It means that most of the cells have undergone late apoptosis (Figure 1B). However, when HSC-2 cells were cultured for 48 hours under the same condition without areca nut treatment, we found that only less than 40% of the cells were viable. [G2] This condition suggests that the preparation of the staining process[G3]  in flow cytometry itself may trigger the death of the cells (apoptosis or necrosis). This includes one of the limitations of our research.[G4] [G5] [G6]  The same result is seen in the HSC-3 cells for 24 hours without treatment.***Figure 3B and 4B:By changing M1 and M2 to caspase-3 (-) and caspase-3 (+), respectively, readers will be able to understand the results more easily. 

*Comments:*

- *Thank you very much for the correction, we have made the corrections as you instructed*
- Discussion page 10, the middle of 1st para:The authors mentioned "the percentage of HSC-2 cells undergoing apoptosis is high than HSC-3 cells. This result is possibly because there is a difference of cell sensitivity againt areca nut extract". However, HSC-2 cells were treated with a higher concentration (629.5 ug/ml) of areca nut extract than HSC-3 cells (164.05 ug/ml). Because the IC50 of HSC-2 cells is higher than that of HSC-3 cells, HSC-3 cells appear to be more sensitive to areca nut extract than HSC-2 cells. This is confusing. The authors should clarify this point.

*Comments:*

- *We have read a report from the previous article :*
- *1. Kamiya Y, Ohshima T. The individual cell properties of oral squamous carcinoma and tumor suppressor gene mutation. Oral Sci Intl 2005;2(2):104-17.2. Sakai* *E,* *and Tsuchida N. Most human squamous cell carcinoma in the oral cavity contain mutated p53* *tumor suppressor* *genes.* *Oncogen* *1992;7:927-33. *
- *We think that it could be our limitation in exploring the characteristics of the HSC-3 cells, but maybe this explanation can open our mind about the result:*
- *This result is possible because of the characteristic of HSC-3 cells is different from HSC-2 cells. The HSC-3 cells have p53 gene mutation. ^3^ The mutation of HSC-3 cells was confirmed in a previous report. ^4^ However, when the p53 gene mutates, the mutated p53 protein is excessively produced or accumulated, thereby compromising apoptosis and leading to abnormal or malignant cell growth. ^5^ We found that HSC-3 cells have the ability to withstand apoptosis higher than HSC-2 cells. However, this finding may vary by the study design and so much more data must be collected to better understand* *this phenomena* *.*

# Peer review: Reviewer response for version 1

**DOI:** 10.5256/f1000research.16170.r36771

**Published:** 2018-08-20

**Authors:** Irna Sufiawati, Solachuddin Jauhari Arief Ichwan, Diah Savitri Ernawati

**Affiliations:** 1 Department of Oral Medicine, Faculty of Dentistry, Padjadjaran University, Bandung, Indonesia; 2 International Islamic University Malaysia, Selangor, Malaysia; 3 Airlangga University, East Java, Indonesia

The manuscript is well written and interesting to read. While the findings are not entirely new, they warrant continued attention because of the current interest in the apoptosis effects of areca nut for oral cancer treatment. However, I see the following issues that should be clarified/resolved before indexing this paper:

 1. Ethic statements. It is unclear whether the cell line has been authenticated, and the methods make no statement that an ethics committee or institutional review board approved the study, which involves the use of human cell lines. If the authors received ethical approval, please include the name of the ethics committee and the approval number.

 2. In the results section, the figures should be made clearer using the appropriate graphing software. 

 3. The discussion section needs to be elaborated. While the discussion includes references to the previous studies, it has not been discussed whether the findings of the study corroborate or contradict those of similar previous studies. In addition, it would be better if the authors discuss the important signaling elements MAPK's and NFκB signaling pathways related to areca nut extract in cancer cells. The discussion appears to be redundant with the results and conclusion section. The discussion also lacks information regarding the limitations and implications of the study.

 4. There is no acknowledgment section in the manuscript. The author should acknowledge anyone who contributed to the study but did not meet the authorship criteria.

 5. There are grammatical errors in the manuscript. The language needs to be improved.

We confirm that we have read this submission and believe that we have an appropriate level of expertise to confirm that it is of an acceptable scientific standard, however we have significant reservations, as outlined above.

## References

[1] : Potential of apoptotic pathway-targeted cancer therapeutic research: Where do we stand?. *Cell Death Dis*.2016;7: 10.1038/cddis.2015.275 e2058 10.1038/cddis.2015.275 26775709PMC4816162

[2] : Effects of arecoline on proliferation of oral squamous cell carcinoma cells by dysregulating c-Myc and miR-22, directly targeting oncostatin M. *PLoS One*.2018;13(1) : 10.1371/journal.pone.0192009 e0192009 10.1371/journal.pone.0192009 29385191PMC5791990

# Response

**Published:** 2018-08-29

**Authors:** Liza Sari

**Affiliations:** Dentistry Faculty Syiah Kuala Indonesia, Indonesia

1. Response:
  - This research has passed the ethics approval with number 501/H2.F1/Etik/2014. The Ethic committee is  The Health Research Ethics Committee of the Faculty of Medicine, University of Indonesia. The chairman is Prof. Dr. dr. Rianto Setiabudy, Sp.FK. (The ethical approval is in the attachment).
  - The HSC-3 and HSC-2 cell lines used in this study were provided by the Oral Biological Laboratory, Faculty of Dentistry of the University of Indonesia. We would like to thank Assoc Prof Masa-Aki Ikeda advisor in Japan who has provided them.
2. We have sent all graphs of the flow cytometry, which are the original results of manual gating using CellQuest software (Becton Dickinson, NJ) to the F1000 Research editorial team. All the graphs contained in the article are the same graphs as the results of flow cytometry but have been saved in JPEG.
3. Response:
  - The study of apoptotic and caspase-3 activities of areca nut on the oral cancer cells, especially HSC-2 and HSC-3, was the novel or first research conducted as far as we know.  The Previous study only has indeed tested ethanolic extract of areca nut cytotoxicity activity against the different type of cancer cells such as MCF-7 cells, and they didn’t count the number of cells undergoing apoptosis, so we don't have any information about other studies with the same form of this research. We also have studied the capability of areca nut in cytotoxicity activity on oral cancer cells and has been published in the other previous journal. We try to be very careful in comparing this study with other previous research especially if they were using different cell types and methods. 
  - Limitations of the study: The apoptosis activity using flow cytometry have several advantages, including fast period time analysis (thousand of cells per second), single cell analysis, and multiparametric measurements (correlations with several different cell events in one unit of time), but this machine also has drawbacks; the presence of physical and enzymatic manipulations during cell preparation and staining, can trigger additional apoptosis or necrosis cell numbers. Furthermore, flow cytometry is only used to calculate the number of apoptotic cells based on PS staining out of the cell membrane. That's why flow cytometry is more appropriate to detect early apoptosis. If the test aims to improve the accuracy of DNA fragmentation calculations in late apoptosis, it's recommended to use *Terminal deoxynucleotidyl transferase (TdT) dUTP Nick-End Labeling* ( *T* *UNEL* **). **
  - The previous study has indeed shown the existence of cell death through extrinsic pathways due to the inhibition of *NF-* *k* *B and MAPKs* by catechin derived from green tea. The discussion in this article is focused on caspase-3 activity as a determinant of the cell death. This study is part of a series of areca nut research that is still going on. The discussion of these proteins will be discussed in our next research.
4. Acknowledgment: This study was a research without grants.
5. This article was translated by Transmedical Institute. Proofreading process was done by Transmedical Institute. The writing technique has been corrected by Grammarly and during the revision process, the editorial team of F1000 research has also improved the sentence structure in the article. 
